# *Capillaria hepatica*—A Neglected Zoonotic Parasite

**DOI:** 10.3390/vetsci13010100

**Published:** 2026-01-20

**Authors:** Juntao Liu, Ruoyan Liu, Jingfei Huang, Qing Liu, Jiarun Cui, Huimei Yu

**Affiliations:** 1 Department of Pathophysiology, College of Basic Medical Sciences, Jilin University, Changchun 130021, China; jtliu2720@mails.jlu.edu.cn (J.L.);; 2 School of Public Health, Jilin University, Changchun 130021, China

**Keywords:** *Capillaria hepatica*, *Calodium hepaticum*, hepatic capillariasis, therapy, diagnosis

## Abstract

As an important zoonotic parasite, *Capillaria hepatica* poses a threat to human health that cannot be ignored due to its association with high mortality and serious damage to the liver, although there are relatively few human infections. The infection rate of *Capillaria hepatica* in rodents is very high, which poses a great threat to the health of rodents, and *Rattus norvegicus* has been found to be the main group carrying *Capillaria hepatica*. *Capillaria hepatica*’s unique biological characteristics, including its morphological features and complex life history, determine the specificity of its infection and pathogenicity. In terms of epidemiology, *Capillaria hepatica* has a worldwide distribution, a wide variety of hosts (mainly rodents), and various transmission routes, all of which increase the difficulty of its prevention and control. Children are more likely to be infected by it, and there is little gender difference among the infected population. Although there are a variety of diagnostic methods for hepatic capillariasis, all of them have certain limitations. In addition, due to its non-specific clinical manifestations, early accurate diagnosis of hepatic capillariasis is still a challenge. This article reviews the biological characteristics and pathogenic mechanism of *Capillaria hepatica*, the epidemiology of human infection, the epidemiology of animal infection, and the diagnosis and treatment of hepatic capillariasis, so as to provide a useful reference for related research and clinical practice.

## Simple Summary

*Capillaria hepatica* is an important but overlooked zoonotic parasite. Infection with *Capillaria hepatica* can lead to severe liver disease and even death. Due to its non-specific clinical manifestations and the difficulty of its diagnosis, its actual incidence is seriously underestimated. In this study, the epidemiology of *Capillaria hepatica* infection in humans and animals is carefully investigated, and the pathogenesis, diagnosis, and treatment of hepatic capillariasis are described in detail. This article provides readers with insights into the unique life cycle and biological characteristics of this parasite, which may help to improve the understanding and control of this parasitic disease.

## 1. Introduction

*Capillara hepatica* (syn. for *Calodium hepaticum*) is a parasitic nematode belonging to the suborder Trichinellina and the family Capillaridae, and its alternative names are *Trichocephalus hepaticus* and *Hepaticola hepatica* [1]. As a zoonotic parasite, the definitive host of *Capillaria hepatica* is mainly rodents, but it can also infect humans [2]. *Capillaria hepatica* parasitizes in the human liver, leading to hepatic capillariasis. The clinical manifestations of hepatic capillariasis lack specificity, and common symptoms include fever, fatigue, loss of appetite, nausea, vomiting, abdominal pain, and diarrhea [3]. Some patients may present with liver involvement, such as hepatomegaly, pain in the liver region, and jaundice. Severe infection may result in liver fibrosis or liver failure [4]. In addition, *Capillaria hepatica* infection can also cause pulmonary symptoms such as cough, expectoration, and chest pain, which may be related to the ectopic parasitic migration of larvae into the lungs [5,6]. Due to its diverse and atypical clinical manifestations, hepatic capillariasis is often misdiagnosed as other liver diseases, such as viral hepatitis, bacterial liver abscess, and liver tumors. The disease is easily overlooked and misdiagnosed due to its low prevalence and lack of specific clinical manifestations. However, the mortality rate of the disease is very high [3]. Hepatic capillariasis is characterized by a global scattered distribution, and related cases have been reported in many countries and regions, and in recent years, there are still related cases reported in India, China, Belgium, and other countries [7,8]. Due to the need for liver biopsy for the definitive diagnosis of hepatic capillariasis, large-scale and systematic epidemiological investigations on hepatic capillariasis are still lacking. In addition, the non-specific clinical manifestations of hepatic capillariasis lead to many mild cases of infection being overlooked and misdiagnosed, which results in a relatively small number of relevant cases for record-keeping and reporting. For these reasons, *Capillaria hepatica* has been neglected by researchers and medical professionals.

In recent years, with the acceleration of global urbanization and the complexity of human settlement, the interaction between humans, animals, and the environment has become increasingly close, which makes the concept of “One-Health” more and more important in the field of public health. More animals share living space with humans, and as important reservoirs and vectors of many emerging and re-emerging zoonotic diseases, they pose potential threats to human health. The natural hosts of *Capillaria hepatica* are mainly rodents, and the infection range of this parasite has been extended to a variety of animals (such as cats, dogs, rabbits, etc.) and humans through predation or environmental contamination (such as contaminated food and water) [9,10]. Studies have shown that poor hygiene and frequent contact with rodents increase the risk of infection with *Capillaria hepatica* [11]. This study reviews all human infection cases and animal infection cases recorded so far, aiming to provide a useful reference for related research and clinical practice. A thorough understanding of the biological characteristics, pathogenic mechanism, epidemiological situation, diagnostic methods, and control measures for *Capillaria hepatica* is of great practical significance for the protection of human health and the prevention of disease transmission.

## 2. General Characteristics and Pathogenic Mechanisms of *Capillaria hepatica*

**Figure 1:**
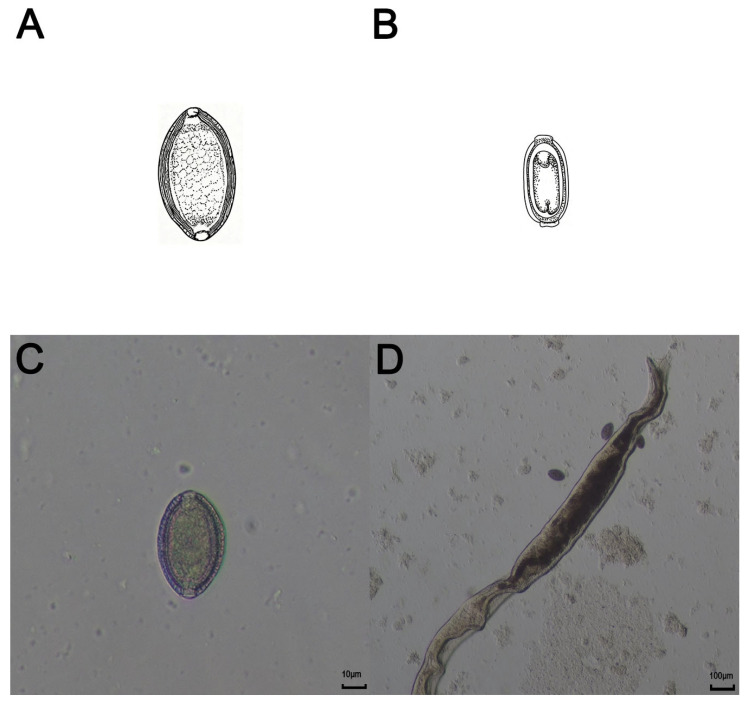
(**A**) Hand-drawn schematic of an egg of *Capillaria hepatica*. (**B**) Hand-drawn schematic of an egg of *Capillaria filipina.* (**C**) Light microscope images of an egg of *Capillaria hepatica*. Scale bars = 10 μm. (**D**) Light microscope image of adult *Capillaria hepatica*. Scale bars = 100 μm.

The eggs of *Capillaria hepatica* are brown and oval, and contain two shell layers; the shell is relatively thick, and the outer shell is depressed. Its eggs are very similar to those of *Capillaria filipina*, but the eggs of *Capillaria hepatica* are larger than those of *Capillaria filipina*, with sizes of 48–66 μm × 28–36 μm and 36–45 μm × 17–21 μm (Figure 1A,B) [12,13]. Moreover, the horizontal lines on the egg shell surface of *Capillaria hepatica* are clearer than those of *Capillaria filipina*, and the former is oval in shape, while the latter is long, oval, and peanut-shaped, and the middle of an egg of *Capillaria filipina* is slightly narrow [13]. The adult body of *Capillaria hepatica* is slender, the anterior part of the body is narrow, the posterior part is enlarged and thick, and the end is blunt and round (Figure 1D). The size of the female nematode is 53–78 mm × 0.11–0.20 mm, and the size of the male nematode is 24–37 mm × 0.07–0.10 mm. The male nematode has copulatory spines, which are 0.43–0.50 mm long [14].

**Figure 2:**
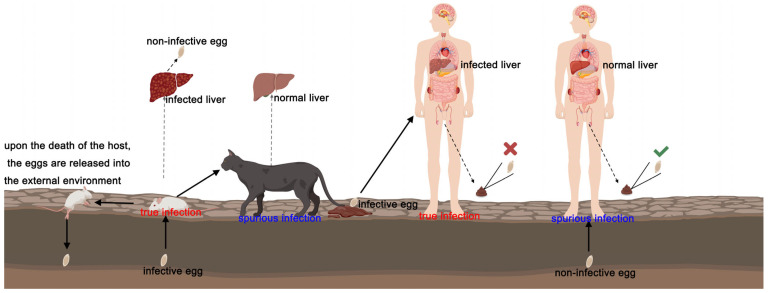
Biological cycle of the *Capillaria hepatica*. The figure was created with the research platform “Med Peer” (medpeer.cn). The “cross mark” indicates that there are no *Capillaria hepatica* eggs in human feces, and “tick mark” indicates that there are *Capillaria hepatica* eggs in human feces. Spurious infection is the ingestion of immature eggs or the ingestion of livers containing non-infective eggs of *Capillaria hepatica* that are only passed through the digestive tract in the feces of the host. True infection refers to the ingestion of infectious eggs, which develop in the host body and reproduce in the liver, but no eggs are excreted in the host’s feces. When a liver containing eggs is eaten by other hosts, the eggs are released under the action of digestive fluid and excreted with the host feces. Alternatively, after the an animal with real infection dies and decays, the eggs are released from the body and develop into infective eggs in a suitable external environment. The host is infected by eating food or water contaminated with infective eggs, the infective eggs develop in the host body, and the adult *Capillaria hepatica* live in the liver and reproduce there.

The eggs of *Capillaria hepatica* develop in soil and are highly resistant to the outside world. Most eggs can tolerate freezing for 1–2 weeks and still survive at −15 °C in winter. The eggs need appropriate temperature, humidity, and oxygen to develop in the outside world. The eggs can develop and become infectious in about 4 weeks at 30 °C and 7 weeks at 23 °C. The host is infected by swallowing food or water contaminated with the infective eggs. Twenty-four hours later, the larvae invade the intestinal mucosa, pass through the mesenteric vein and portal vein, and then reach the liver, where they grow and develop [15]. The lifespan of females is about 59 days, and that of males is about 40 days [16]. After 28 days of infection, pregnant females can be found, and the eggs produced by females are mostly collected in the liver and rarely excreted. At this time, the eggs in the liver tissue do not develop into infective eggs [17]. After the death of the host, the corpse decays, and the eggs in it re-enter the external environment and develop into infectious eggs, or the infected host is preyed on by other hosts, and the eggs are discharged into the external environment with the predator’s feces, and then develop into infective eggs [18]. Therefore, *Capillaria hepatica* is a nematode that requires the death of the host to complete its life cycle [19]. A schematic of the biological cycle of the *Capillaria hepatica* can be seen in Figure 2. Infections caused by *Capillaria hepatica* can be divided into spurious infections and true infections. Spurious infection is the ingestion of immature eggs or the ingestion of livers containing non-infective eggs of *Capillaria hepatica* that are only passed through the digestive tract in the feces of the host. True infection refers to the ingestion of infectious eggs, which develop in the host body and reproduce in the liver, but no eggs are excreted in the host’s feces; this type of host is called a definitive host of *Capillaria hepatica* [20]. After invading the liver tissue, *Capillaria hepatica* will release excretory/secretory proteins (ESPs) involved in immune regulation to avoid being killed by the host immune system, which makes it difficult to find them in the early stage of infection [21]. The continuous growth and development of *Capillaria hepatica* in the liver will cause hepatosplenomegaly, and its degree is consistent with the number of eggs in the liver. Many white or gray-yellow nodules on the surface of the liver can be seen by the naked eye, sometimes fused into a hard irregular mass. In addition, epithelial cell granulomatous reactions involving multinucleated giant cells and eosinophilic inflammatory infiltration have been observed around the eggs [22]. At the same time, it induces the expression of liver fibrosis-related proteins, including laminin, collagen IV, procollagen III, and hyaluronic acid, leading to liver fibrosis [23].

## 3. Epidemiological Overview and Preliminary Estimation of Hepatic Capillariasis in Human Population

**Figure 3:**
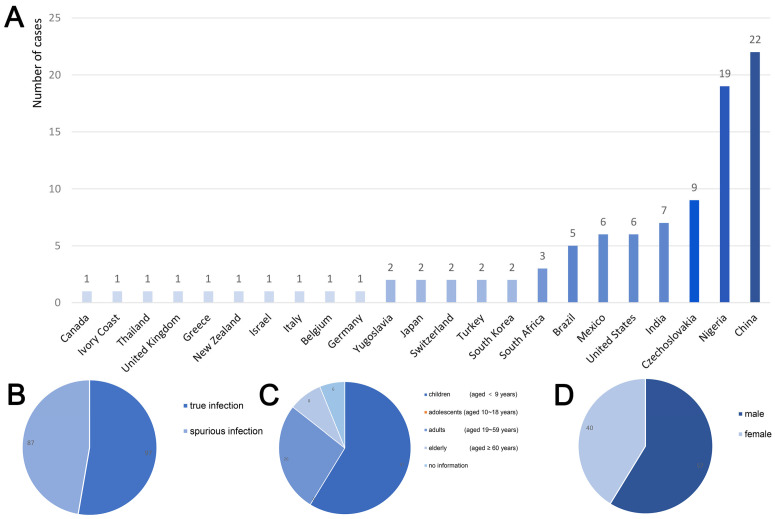
(**A**) Bar graph of regional distribution of hepatic capillariasis. X-axis: countries; Y-axis: the number of cases. The darker the blue, the higher the number of cases. (**B**) Fan plot of true and spurious infections with hepatic capillariasis. (**C**) Fan plot of the age distribution of hepatic capillariasis. (**D**) Fan plot of the sex distribution of hepatic capillariasis. All data presented in this figure are from [1,7,8,23,28,29,30,31,32,33,34,35,36,37].

Hepatic capillariasis exhibits a globally scattered distribution pattern. Human infection cases have been reported across Europe (including Germany, Switzerland, Italy, the United Kingdom, Greece, former Czechoslovakia, former Yugoslavia, Turkey), the Americas (the United States, Canada, Mexico, and Brazil), Asia (India, South Korea, Japan, Thailand, and regions such as Guangdong, Henan, and Fujian in China), Africa (South Africa, Côte d’Ivoire, and Nigeria), and Oceania (New Zealand), as shown in Figure 3A. As a zoonotic pathogen with rodents as the main reservoir hosts, its transmission is closely related to the population density and activity range of rodents. However, unlike other parasites, it has not established highly concentrated endemic zones globally, with sporadic cases predominantly reported worldwide. Infection can be classified into pseudo-infection and true infection. Based on papers obtained from electronic databases (Scopus, PubMed, and Google Scholar), this study reviewed a total of 197 recorded human infection cases to date, among which 49.24% (97/197) were true infections (Figure 3B). Due to the lack of large-scale systematic epidemiological investigations and the heavy reliance on invasive diagnostic methods such as liver biopsy, many mild or asymptomatic infections may not have been detected, making it difficult to accurately estimate the actual number of global infections [23].

Multiple factors jointly influence the infection risk and prevalence of *Capillaria hepatica*. In terms of age, children show higher susceptibility, as shown in Figure 3C, with 58.8% of the reported patients being under 9 years old. This is closely related to children’s behavioral habits, such as crawling on the ground, sucking fingers, and accidentally ingesting contaminated items. Investigations of gender differences reveal a higher proportion of male infections (Figure 3D), potentially associated with higher exposure risks, differing hygiene practices, and high-risk dietary habits among males. Other studies have identified that immune function and sanitary conditions are key influencing factors: individuals with compromised immunity and impoverished populations living in damp or contaminated environments face heightened infection risks. Drinking unboiled contaminated water directly increases the probability of ingesting infective eggs [24]. Additionally, the density and activity range of reservoir hosts such as rodents determine the environmental distribution of eggs, constituting an important ecological basis for disease transmission [25,26].

As shown in Figure 3A, there are significant differences in the infection status of *Capillaria hepatica* among different countries and regions. These differences are essentially the result of the combined effects of environmental factors, behavioral patterns, and public health standards. Currently, the global understanding of the epidemiological patterns of hepatic capillariasis is still limited to case reports, lacking standardized epidemiological surveillance networks and cross-regional comparative data on prevalence. The actual infection situation may be underestimated. Therefore, it is urgently necessary to conduct systematic global epidemiological investigations, trace the transmission chains using molecular epidemiological techniques, and establish risk prediction models based on rodent population density, sanitation conditions, and dietary behaviors. Concurrently, lessons from other parasitic disease control efforts can be applied. Comprehensive measures—including enhanced rodent control, widespread hygiene education, and avoidance of raw or undercooked foods—can reduce population infection risks [27].

## 4. Hosts of *Capillaria hepatica* and Its Epidemiological Characteristics in Animals

*Capillaria hepatica* is a zoonotic parasite with low host specificity, widely parasitizing the livers of various animals. It has been detected in the livers of nearly 180 mammalian species worldwide [38]. Examples include rodents, horses, porcupines, cats, dogs, rabbits, southern sea otters, and ring-tailed lemurs [9,39,40].

**Figure 4:**
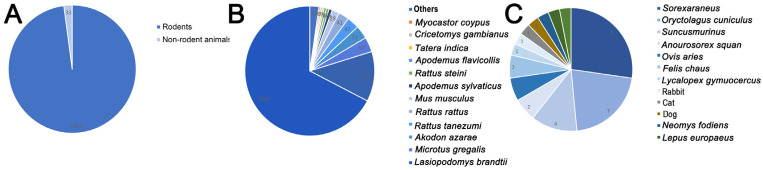
(**A**) Distribution of *Capillaria hepatica* infection cases by species in animals from 2014 to 2025 (1574 observed outbreaks). (**B**) Distribution of *Capillaria hepatica* infection cases by species in rodents from 2014 to 2025 (1541 observed outbreaks). Others: including rodents with fewer than 5 cases of infection. (**C**) Distribution of *Capillaria hepatica* infection cases by species in non-rodent animals from 2014 to 2025 (33 observed outbreaks). Data from [2,16,41,42,43,44,45,46,47,48,49,50,51,52,53,54,55,56,57,58,59,60,61,62,63,64,65,66,67,68,69,70,71,72,73,74,75,76,77].

As shown in Table 1, this study reviewed the recorded cases of *Capillaria hepatica* infection in animal hosts from 2014 to 2025 in open-access electronic databases (Scopus, Pubmed and Google Scholar). Data prior to 2014 were available in two previous studies [38,41]. The results of this study indicated that rodents are important hosts of *Capillaria hepatica*, which was consistent with previous research findings. Among all infected animals, rodents accounted for 97.9%, while non-rodents only accounted for 2.1% (Figure 4A). Rodents played a key role in the spread and distribution of *Capillaria hepatica*, which had a wide distribution and was most prominent in specific rodent species. As shown in Figure 4B, from the perspective of host categories, *Rattus norvegicus* was the main group carrying *Capillaria hepatica*. Studies have shown that there are significant differences in the carrying rates among different rodent species. The prevalence of *Capillaria hepatica* in *Rattus norvegicus* is widespread in multiple countries and regions. In one study, 144 *Rattus norvegicus* were captured, and the carrying rate of *Capillaria hepatica* was 44.44% [42].

The prevalence of hepatic capillariasis in rodents has a significant impact on disease transmission and public health security. The eggs of the parasite that are scattered in the environment after the death of rodents can be ingested by other animals or humans through food, causing hepatic capillariasis and establishing a transmission chain of the parasite in nature and among various hosts, thus facilitating cross-host transmission and spread. In crowded and polluted environments, over 70% of rodents are infected with hepatic capillariasis, and the cannibalism among rodents of the same species promotes the intergenerational transmission of the disease, further expanding the infection range [38,43]. Therefore, understanding the species of rodents and the prevalence of hepatic capillariasis in these species in specific regions can provide a basis for scientifically and precisely formulating prevention and control strategies, reducing the possibility of disease outbreaks.

**Table 1:** The infection situation of *Capillaria hepatica* in animals (2014–2025).

Species of Host	Infection Rate	References
***Rodents***		
*Akodon azarae*	51/145	[60]
*Apodemus flavicollis*	9/188	[49,63]
*Apodemus sylvaticus*	13/322	[48]
*Arvicanthis niloticus*	4/89	[59]
*Arvicolaamphibius*	1/1	[49]
*Bandicotaindica*	1/3	[70]
*Calomys callidus*	4/29	[60]
*Calomys venustus*	1/101	[60]
*Clethrionomys glareolus*	1/84	[49]
*Cricetulus migratorius*	1/5	[58]
*Cricetomys gambianus*	7/40	[57,64]
*Lasiopodomys brandtii*	192/1002	[69]
*Meriones persicus*	2/114	[67]
*Meriones vinogradovi*	1/10	[67]
*Microtus agrestis*	1/8	[49]
*Microtus gregalis*	57/267	[68]
*Mus musculus*	28/378	[44]
*Myocastor coypus*	6/46	[61,62]
*Niviventer coninga*	1/4	[70]
*Niviventer fulvescens*	2/9	[70]
*Oligoryzomys flavescens*	3/51	[60]
*Oligoryzomys nigripes*	1/15	[60]
*Oxymycterus rufus*	3/47	[60]
*Rattus andamanensis*	1/9	[42]
*Rattus argentiventer*	1/27	[65]
*Rattus exulans*	1/7	[2]
*Rattus norvegicus*	1038/2382	[2,42,43,44,45,46,47,48,49,50,51,52,53,54]
*Rattus nitidus Hodgson*	1/7	[70]
*Rattus rattus*	41/104	[18,51,55,56,57]
*Rattus steini*	10/27	[70]
*Rattus tanezumi*	47/437	[2,42,54,65,70,71]
*Rattus yunnanensis*	4/10	[70]
*Tatera indica*	7/33	[66]
**Non-rodent animals**		
*Anourosorex squam*	2/56	[70]
*Cat*	1/1	[72]
*Dog*	1/1	[9]
*Felis chaus*	2/2	[75]
*Horse*	1/1	[78]
*Lepus europaeus*	1/1	[73]
*Lycalopex gymnocercus*	1/1	[76]
*Neomys fodiens*	1/3	[49]
*Oryctolagus cuniculus*	7/87	[39]
*Ovis aries*	2/110	[74]
*Rabbit*	1/1	[77]
*Sorexaraneus*	9/60	[49]
*Suncusmurinus*	4/110	[65]

Infection rate: total number of infected animals/total number of captured animals.

## 5. Diagnosis of Hepatic Capillariasis

### 5.1. Pathological Examination

Liver biopsy is the gold standard for diagnosing *Capillaria hepatica* infection [23]. Liver tissue specimens obtained via liver puncture or surgery undergo pathological examination, with the presence of eggs or adult worms confirming the diagnosis. Pathological examination reveals granulomatous inflammation within liver tissue, featuring eggs or adult worms at the center surrounded by extensive inflammatory cell infiltration and fibrosis [14]. However, liver biopsy is an invasive procedure carrying risks such as bleeding and infection. Additionally, some patients may yield false-negative results due to uneven lesion distribution. Therefore, in clinical practice, the decision to perform a liver biopsy requires comprehensive consideration of the patient’s specific circumstances and careful weighing of the benefits and risks.

### 5.2. Immunological Examination

Immunological tests for hepatic capillariasis include the immunofluorescence assay and enzyme-linked immunosorbent assay (ELISA) methods, which provide significant diagnostic value. Immunofluorescence assays utilize fluorescently labeled specific antibodies to bind with antigens. Under a fluorescence microscope, the presence of fluorescence indicates whether corresponding antigens exist in the sample. This method offers high sensitivity, aiding in early disease diagnosis, but its specificity is relatively low. It is prone to cross-reactions with other parasites, potentially yielding false-positive results [79]. ELISA employs enzyme-labeled antigens or antibodies to bind with corresponding antigens or antibodies in the sample. Through enzyme-catalyzed substrate color development, it detects the presence of target substances. This method is sensitive, highly specific, and easy to conduct [1]. However, antigen cross-reactions may occur, potentially affecting diagnostic accuracy. Therefore, in clinical practice, positive immunological test results require comprehensive evaluation in conjunction with the patient’s clinical presentation, epidemiological history, and other test findings to avoid misdiagnosis.

### 5.3. Imaging Examination

Magnetic resonance imaging (MRI), computed tomography (CT), and ultrasound are crucial diagnostic tools for hepatic capillariasis, aiding in the detection of hepatic lesions. MRI is considered the preferred method for evaluating parasitic lesions, as it can detect more complex morphological changes and infiltrations [23,80]. Ultrasound is non-invasive and convenient, allowing real-time observation of liver morphology and structure. In patients with liver filariasis, preliminary assessment typically reveals heterogeneous liver echogenicity, ill-defined borders, and multiple hypoechoic or hyperechoic nodular masses with indistinct margins [1]. However, smaller or deep lesions may be difficult to visualize clearly due to patient body type and intestinal gas. CT offers higher resolution, enabling clear depiction of fine liver structures and lesions. In diagnosing hepatic capillariasis, CT scans can reveal hepatomegaly, masses, or cystic lesions [81]. Contrast-enhanced CT further evaluates lesion blood supply, aiding in differential diagnosis. However, CT carries radiation risks and may have limitations in detecting early or mild lesions, necessitating integration with other diagnostic modalities for comprehensive assessment.

In summary, the diagnosis of hepatic capillariasis requires the integrated application of multiple methods, including parasitological examination, immunological examination, and imaging examination, which should complement and corroborate one another.

### 5.4. Differential Diagnosis

*Capillaria hepatica* infections require differential diagnosis from other liver diseases. Compared to viral hepatitis, patients with *Capillaria hepatica* infection typically lack an epidemiological history of viral hepatitis, test negative for viral markers, and exhibit more pronounced eosinophilia [82,83]. Compared to bacterial liver abscesses, patients with *Capillaria hepatica* infection show no significant signs of infection or toxemia, bacterial growth is absent in abscess aspirate, and anti-infective therapy proves ineffective. Compared with hepatic tumors, patients with *Capillaria hepatica* infection generally lack a history of malignancy, exhibit normal tumor markers such as alpha-fetoprotein, and demonstrate distinct imaging features. Additionally, differential diagnosis must be conducted with other parasitic liver diseases, including hepatic echinococcosis and clonorchiasis, primarily based on a comprehensive assessment of epidemiological history, clinical presentation, laboratory findings, and imaging characteristics [84,85,86].

## 6. Treatment of Hepatic Capillariasis

There is no specific drug for hepatic capillariasis. In clinical practice, anthelmintic drugs such as albendazole, mebendazole, and ivermectin are mostly used for treatment. When granulomatous hepatitis caused by egg deposition presents with symptoms such as high fever and hepatosplenomegaly, corticosteroids like glucocorticoids, prednisolone, or prednisolone acetate are required to reduce the inflammatory response [87]. For severe cases, other treatment methods such as surgery may need to be combined.

In pharmacotherapy, albendazole is the preferred broad-spectrum agent. It interferes with parasite microtubule synthesis and inhibits glucose uptake, leading to glycogen depletion and eventual death of the parasite [88]. Liver function and complete blood counts should be monitored during treatment. Some patients experience mild gastrointestinal reactions, which resolve upon discontinuation. Mebendazole can effectively kill parasite eggs and affect the parasite’s metabolism through multiple pathways. Compared with albendazole, it causes fewer adverse reactions but exhibits poor oral absorption [89]. Ivermectin enhances the effect of γ-aminobutyric acid in the parasite, blocks the transmission of nerve signals, and causes the parasite to become paralyzed and die [90]. However, its clinical application is relatively limited. For patients with severe conditions and intense inflammatory responses, combination therapy may be considered. For example, the combination of albendazole and mebendazole can enhance the therapeutic effect, or prednisone can be added to reduce inflammation and alleviate symptoms [1].

For patients with drug-refractory cases or severe complications (such as severe liver tissue destruction, liver failure, biliary bleeding, etc.), partial hepatectomy may be considered to remove the lesions and improve liver function. However, this surgery carries a high risk, and various complications may occur postoperatively. Therefore, it is necessary to strictly define the surgical indications and fully assess the patient’s surgical tolerance. In addition, follow-up data show that some patients develop liver fibrosis during reexamination after receiving standardized antihelminthic treatment [91]. This suggests that in the treatment of hepatic capillariasis, in addition to antihelminthic and anti-inflammatory therapies, early combination with antifibrotic drugs is also required.

The treatment of *Capillaria hepatica* infection faces three key problems, which seriously restrict the efficacy of diagnosis and treatment. First, there is a lack of large-scale clinical data. Most of the current relevant clinical studies are case reports or small-sample studies, and there is a shortage of large-scale, multi-center randomized controlled trials. This leads to insufficient evidence-based medicine for formulating treatment plans, making it difficult to determine the optimal therapeutic drugs, dosages, and courses of treatment. Moreover, there are differences in the level of diagnosis and treatment among different regions and hospitals, and the selection of treatment plans often relies on the personal experience of doctors, resulting in uneven treatment effects. Therefore, it is urgent to carry out large-scale clinical studies to accumulate data and provide a basis for the formulation of standardized treatment plans. Second, there are issues of drug side effects and drug resistance. Anthelmintic drugs may cause side effects such as gastrointestinal reactions, liver function damage, and bone marrow suppression during treatment. Some patients have poor tolerance to the drugs and cannot complete the prescribed course of treatment, which in turn affects the treatment effect [92]. At the same time, long-term use of anthelmintic drugs may lead to the development of drug resistance in parasites. Therefore, during treatment, it is necessary to closely monitor patients’ adverse drug reactions, adjust their treatment plans in a timely manner, and at the same time strengthen the monitoring of drug resistance and use drugs rationally. Third, difficulties in early diagnosis led to delayed treatment. The clinical manifestations of this disease are non-specific, and the diagnostic methods have limitations, making early diagnosis difficult. By the time patients seek medical treatment due to obvious symptoms, in many cases, their condition has already progressed to a severe stage, with severe damage to liver tissue, increasing the difficulty of treatment. Some patients are also misdiagnosed as having other liver diseases. Therefore, improving clinicians’ understanding of this disease and strengthening the research and promotion of early diagnostic technologies are the keys to improving the prognosis of patients.

## 7. Conclusions

At present, some achievements have been made in the research on *Capillaria hepatica*, covering its biological characteristics, epidemiology, diagnostic methods and control measures. In the study of biological characteristics, the morphological characteristics and life history of *Capillaria hepatica* are well understood, and the morphological characteristics of adults and eggs, as well as their development process under different environmental conditions, have been clarified. However, there are still some shortcomings in the current study. In terms of diagnosis, although there are a variety of examination methods, all of them have certain limitations. Liver biopsy is the “gold standard” in etiological examination, but it is an invasive examination with risks and high technical requirements. The specificity and sensitivity of immunological tests need to be further improved. In areas where a variety of parasites are endemic, antigen cross-reaction affects the diagnostic accuracy. Imaging studies have limitations in the diagnosis of early or minor lesions. In the field of treatment, although existing therapeutic drugs such as albendazole and mebendazole are effective, they have side effects, and for some severe cases, the effect of drug treatment is limited. Although ivermectin has a certain effect, its clinical application is relatively rare, and its mechanism of action and efficacy stability need to be further studied.

Future studies should focus on the following directions. In terms of diagnostic methods, efforts should be made to develop more accurate, convenient and non-invasive detection techniques, such as molecular-biology-based diagnostic methods, to improve the specificity and sensitivity of detection and achieve early diagnosis. In terms of treatment, it is necessary to develop safer and more effective new drugs, further study the mechanisms of action of existing drugs, optimize treatment plans, and improve the cure rate. It is necessary to strengthen the surveillance and early warning of hepatic capillariasis, establish a perfect surveillance system, detect epidemics in time, and take effective control measures to reduce the threat to human health.

## Data Availability

The original contributions presented in this study are included in the article. Further inquiries can be directed to the corresponding author.
